# Uncovering psychiatric phenotypes using unsupervised machine learning: A data-driven symptoms approach

**DOI:** 10.1192/j.eurpsy.2023.13

**Published:** 2023-02-21

**Authors:** Amy Hofman, Isabelle Lier, M. Arfan Ikram, Marijn van Wingerden, Annemarie I. Luik

**Affiliations:** 1 Department of Epidemiology, Erasmus MC University Medical Center Rotterdam, Rotterdam, The Netherlands; 2 Department of Cognitive Science and Artificial Intelligence, Tilburg University, Tilburg, The Netherlands; 3 Department of Child and Adolescent Psychiatry/Psychology, Erasmus MC University Medical Center Rotterdam, Rotterdam, The Netherlands

**Keywords:** Anxiety disorders, depression, machine learning, sleep–wake disorders

## Abstract

**Background:**

Current categorical classification systems of psychiatric diagnoses lead to heterogeneity of symptoms within disorders and common co-occurrence of disorders. We investigated the heterogeneous and overlapping nature of symptom endorsement in a population-based sample across three of the most common categories of psychiatric disorders: depressive disorders, anxiety disorders, and sleep–wake disorders using unsupervised machine learning approaches.

**Methods:**

We assessed a total of 43 symptoms in a discovery sample of 6,602 participants of the population-based Rotterdam Study between 2009 and 2013, and in a replication sample of 3,005 participants between 2016 and 2020. Symptoms were assessed using the Center for Epidemiologic Studies Depression Scale, the Hospital Anxiety and Depression Scale, and the Pittsburgh Sleep Quality Index. Hierarchical clustering analysis was applied on test items and participants to investigate common patterns of symptoms co-occurrence, and further quantitatively investigated with clustering methods to find groups that may represent similar psychiatric phenotypes.

**Results:**

First, clustering analyses of the questionnaire items suggested a three-cluster solution representing clusters of “mixed” symptoms, “depressed affect and nervousness”, and “troubled sleep and interpersonal problems”. A highly similar clustering solution was independently established in the replication sample. Second, four groups of participants could be separated, and these groups scored differently on the item clusters.

**Conclusions:**

We identified three clusters of psychiatric symptoms that most commonly co-occur in a population-based sample. These symptoms clustered stable over samples, but across the topics of depression, anxiety, and poor sleep. We identified four groups of participants that share (sub)clinical symptoms and might benefit from similar prevention or treatment strategies, despite potentially diverging, or lack of, diagnoses.

## Introduction

The current categorical classification systems of psychiatric diagnoses, such as the Diagnostic and Statistical Manual of Mental Disorders (DSM) or International Classification of Diseases (ICD) frameworks, have demonstrated reliable categories of diagnoses [1, 2], however, these frameworks have also been shown to suffer from important limitations. For example, psychiatric disorders share underlying processes regarding etiology and maintenance, which has made it difficult to identify both general and specific biological mechanisms underlying these disorders [3–5]. Additionally, diagnosis is complicated by the heterogeneity of symptoms within disorders and overlapping symptoms between disorders leading to common co-occurrence [6]. As these disorders often share characteristics and symptoms, the large number of disorder-specific treatment protocols and manuals in clinical practice may not be necessary [3–5].

Multiple transdiagnostic frameworks have been proposed to overcome these limitations. For example, the Research Domain Criteria (RDoC) framework was developed which assesses transdiagnostic psychiatric phenotypes as a manifestation of different functional and psychobiological domains [7]. Yet, this framework was mainly developed for research purposes and can to date not be translated to clinical practice. In contrast, the Hierarchical Taxonomy of Psychopathology (HiTOP) aims to provide clinical applications based on a dimensional approach of psychiatric symptoms [8, 9]. However, a systematic review of transdiagnostic approaches has shown that these approaches have so far not led to a paradigm shift that leads to changes in classification of psychiatric disorders [3].

Bottom-up data-driven approaches might be promising, as they could provide insights in patterns of symptom co-occurrence. Unsupervised machine-learning methods are a class of approaches that seek to uncover patterns or clusters in high-dimensional datasets, and thus could be used to uncover alternative symptom and participant groupings. The HiTOP framework was developed based on such approaches, resulting in dimensions at multiple levels (e.g., “internalizing” at the spectrum level and “fear” and “distress” as subfactors within this spectrum) [8, 9]. However, the majority of studies could not assess complete symptom profiles, as they are often based on diagnostic interviews that include skip logic. Also, evidence used for the HiTOP framework was mainly based on adults aged 18–65 years old. Studies using a symptom-based approach, using questionnaires that assess complete symptom profiles, are particularly needed in middle-aged and older adults since subclinical symptoms are common, and can be experienced as debilitating [10–12]. By focusing on the occurrence of symptoms from different rating scales in the general population, patterns can be highlighted that span the continuous spectrum of symptom presentations, providing avenues for joint prevention and treatment strategies.

Lastly, previous studies were mainly based on factor analysis or class-based methods. However, maximum-likelihood factor analyses are less appropriate for studying subclinical symptoms in a population-based sample as the responses on all symptom items show a heavily skewed distribution due to querying of clinical symptom endorsement in a mostly nonclinical sample. To avoid using a (Pearson) correlation structure as the basis of symptoms clustering, hierarchical clustering analyses (HCAs) with the linkage criteria appropriate for binary data are preferred [13, 14].

Therefore, we investigated the heterogeneous and overlapping nature of symptoms in the population-based Rotterdam Study across three of the most common categories of psychiatric disorders: depressive disorders, anxiety disorders, and sleep–wake disorders using unsupervised machine learning approaches.

## Materials and Methods

### Study design and participants

This study was embedded within the Rotterdam Study, a prospective population-based cohort study that originated in 1990 in a suburb of Rotterdam, the Netherlands. The goal is to study the occurrence and determinants of age-related diseases in the general population [15]. In total, 17,931 persons aged 40 years and over participated. The overall response rate across the study waves was 66%.

The discovery sample comprised 7,162 participants that took part in the study between 2009 and 2013. Of these, 7,156 participants completed the interview that involved questions on depression, anxiety, and sleep quality. Because of logistic reasons, 554 participants did not complete the questionnaire on sleep quality and had to be excluded, leaving 6,602 participants for analyses. The replication sample consisted of 3,005 participants that participated in the study between 2016 and 2020.

The Rotterdam Study has been approved by the Medical Ethics Committee of Erasmus MC (registration number MEC 02.1015) and by the Dutch Ministry of Health, Welfare, and Sport (Population Screening Act WBO, license number 1071272–159521-PG). The Rotterdam Study Personal Registration Data collection is filed with the Erasmus MC Data Protection Officer under registration number EMC1712001. The Rotterdam Study has been registered at the Netherlands National Trial Register (NTR; www.trialregister.nl) and the WHO International Clinical Trials Registry Platform (ICTRP; www.who.int/ictrp/network/primary/en/) under shared catalog number NTR6831. All participants provided written informed consent to participate in the study and to have their information obtained from treating physicians.

## Measurements

### Depressive symptoms

Depressive symptoms were assessed using the Dutch version of the Center for Epidemiological Studies Depression Scale (CES-D), which was designed to assess presence and severity of depressive symptoms in the general population [16, 17]. The questionnaire consists of 20 items, that were answered on a four-point Likert scale during the home interview. The score ranges between 0 and 60, with a higher score reflecting more depressive symptoms. A score of 16 or above indicates clinically relevant depressive symptoms.

Participants that screened positive for depressive symptoms on the CES-D scale were invited for a semi-structured clinical interview by clinically trained research personnel: the Dutch version of the Schedules for Clinical Assessment in Neuropsychiatry (SCAN) [18]. Based on the interview, depressive disorders were diagnosed and classified according to the Statistical Manual of Mental Disorders, 4th revised edition (DSM-IV-TR) [19].

### Anxiety symptoms

Anxiety symptoms were measured using the Hospital Anxiety and Depression Scale (HADS) [20, 21]. The subscale for anxiety (HADS-A) consists of seven items that were asked during the home interview. These seven items were answered on a four-point Likert scale, resulting in a total score ranging from 0 to 21. A higher score indicates more anxiety symptoms and the cut-off score to determine anxiety symptoms is 8 or higher.

In addition, an adapted version of the Munich version of the Composite International Diagnostic Interview (M-CIDI) was administered during the home interview [22]. The M-CIDI was specifically designed to obtain DSM-IV-TR diagnoses and assesses the 1-year prevalence of generalized anxiety disorder, panic disorder, agoraphobia, social phobia, and specific phobia, according to the DSM-IV-TR criteria [19]. If participants screened positive for one of these disorders, they were classified as having an anxiety disorder.

### Sleep quality

Sleep quality was assessed during the home interview using the Pittsburgh Sleep Quality Index (PSQI) [23]. The PSQI is a self-rating questionnaire that consists of 24 items on sleep quality and disturbance. The total score ranges from 0 to 21, with a higher score reflecting poorer sleep quality.

### Other variables

Information on other variables was collected during the home interview. Self-report data were available for sex and age. Educational level was categorized according to the UNESCO classification into primary, lower, intermediate, and higher education. Marital status was asked and categorized into “living with partner” and “living alone.” Employment status was based on paid employment (“yes” or “no”). History of chronic diseases, that is, cancer, coronary heart disease, stroke, and diabetes, was assessed by self-report and continuous monitoring of medical records. Smoking status was asked and categorized into “current smoker” and “current nonsmoker.”

### Statistical analysis

The study population was characterized using descriptive statistics. Analyses were independently performed in the discovery and replication sample. Missing values on questionnaire items (mean percentage of missings per item: 1.4% in the discovery sample and 1.9% in the replication sample) were imputed by fivefold multiple imputation using the mice package in R [24]. For each missing value on the ordinal questionnaire items, the mode of the five imputed values was obtained to create one dataset for further analyses. Due to the skewed data distributions (most participants indicated no complaints, coded as 0 versus little to severe complaints, coded as 1–3), scores on questionnaire items were dichotomized with “1” replacing any symptom endorsement.

To find the optimal clustering of items and participants, we adopted a two-tier approach (see steps outlined below). Due to the binary nature of the dataset, assumptions for factor analyses or matrix factorization techniques would not be satisfied. We instead opted for agglomerative HCAs as the first approach to the binary data.

HCA is a bottom-up approach, meaning that each data point starts in an individual cluster [25]. The algorithm then merges the closest pair of clusters according to a linkage criterion, in this case, Ward’s method as this method performs well in linking clusters with binary variables [25, 26]. After merging, the distance matrix is updated by computing distances between the new clusters, which is repeated until all clusters are merged into one. HCA was performed independently on test items and participants, as binarized adaptations of simultaneous bi-clustering of test items and participants uses a top-down clustering approach. This yielded small clusters consisting of the two items with the highest number or co-endorsement and thus was less informative on the overall similarity in symptom profiles between participants.

HCA on binarized individual test items was used to find common patterns of symptom co-occurrence and thus transdiagnostic patterns of symptom endorsement [25]. We considered pairs of 1–1 (co-occurring symptoms) as more informative than joint scores of 0 (indicating absence of both symptoms), and therefore data was treated as asymmetrical. Consequently, the similarity in co-occurrence of symptoms was evaluated with the Jaccard index to compute the distance matrix [27]. The results were interpreted using dendrograms (i.e., which items cluster at higher/lower levels in the dendrogram) for the total population, and for men and women separately.

Second, HCA was applied to find groups of participants that were most similar and therefore are likely to represent participants with similar phenotypes. We performed the analysis on individual item scores to ensure the clustering solution was not directly dependent on the clustering of test items. Here, binary data was treated as symmetrical (1–1 and 0–0 score pairs both contain important information) and therefore, the simple matching coefficient was used as proximity measure to compute the distance matrix [28]. These one-dimensional HCA analyses served to determine a region of interest in the number of item cluster (*k*_i_) by number of participant clusters (*k*_p_) space for further exploration and quantification. To assess which number of clusters provided the most parsimonious representation of the data, the following strategy was applied:

Step 1: Visual inspection of the elbow method after running HCA on the binary item and participant data independently (*k* = 2 up to *k* = 20).

Step 2: Based on step 1, create datasets of possible combinations containing aggregated item-cluster scores of *k*_i_ = 3 up to *k*_i_ = 10 item clusters for *k*_p_ = 3 up to *k*_p_ = 10 participant clusters and assess average silhouette width of obtained clusters.

Step 3: Based on step 2, identify region of interest: *k*_i_ = 3 up to *k*_i_ = 5 item clusters for *k*_p_ = 3 up to *k*_p_ = 5 participant clusters. Assess cluster quality of all solutions in this *k*_i_ × *k*_p_ space by running *k*-means and pam clustering on aggregated item cluster scores based on silhouette score and sum of squares measures. These standardized cluster metrics are preferred as these can be compared between datasets with different number of features.

Stability of the identified item clusters was assessed in the replication sample and separate subsets of the dataset, split for males and females. In addition, a resampling method was applied where test items were randomly assigned to the same number of item clusters and submitted to *k*-means, to check whether the HCAs-derived cluster solution performed better than random clustering, evaluated with the between/total sum-of-squares ratio. For our final presentation, we investigated the distribution of cluster scores across participant groups, by summing the scores for each symptom (0/1) within a symptom cluster for each participant, and visualized these using boxplots.

As *trans*-symptomatic clustering might have been hampered by the skewed distribution of data (high number of participants reporting 0 or few symptoms), we created a subset of the data including participants with a known diagnosis of depression (i.e., major depressive disorder or dysthymia, *N* = 40), or any anxiety disorder (*N* = 352), or both (*N* = 21). As symptom cluster scores are expected to better approximate a normal distribution in this subsample, Gaussian finite mixture modeling was used as clustering approach, and Bayesian Information Criterion (BIC) values were compared to obtain the optimal number of clusters and covariance structure of the data (MClust package in R [29]). To control for potential bias in clustering based on diagnosis, a second clustering with undersampling of the majority class (anxiety diagnosis; *N* = 373/434) was performed.

## Results

Characteristics of the discovery sample are presented in Table 1. Of the discovery sample (*N* = 6,602, mean age: 69.5, standard deviation [SD]: 9.4 years), the majority was female (57.8%), and living with partner (68.9%). The replication sample (*N* = 3,005) was younger (mean age: 57.3, SD: 11.4 years) and more often higher educated (30.4%) and underpaid employment (61.8%, Table 1 in the Supplementary Material).

**Table 1.** Characteristics of the study sample.

	Discovery sample (*N* = 6,602)
Age (years)	69.5 (9.4)
Sex	
Female	3,816 (57.8%)
Educational level	
Primary	579 (8.8%)
Lower	2,652 (40.2%)
Further/intermediate	1,915 (29.0%)
Higher	1,388 (21.0%)
Marital status	
Living with partner	4,545 (68.9%)
Living alone	2,048 (31.1%)
Paid employment	
Yes	1,363 (21.5%)
History of cancer	719 (10.9%)
History of coronary heart disease	608 (9.3%)
History of stroke	285 (4.3%)
History of diabetes	778 (11.9%)
Smoking	
Current smoker	848 (12.9%)
Current nonsmoker	5,733 (87.1%)
Depressive symptoms (CES-D) *median(IQR)*	3.0 (1.0–8.0)
Anxiety (HADS) *median(IQR)*	2.0 (0.0–4.0)
Sleep quality (PSQI) *median(IQR)*	3.0 (1.0–6.0)

*Note*: Data are presented as mean (SD) or *N* (%) unless otherwise indicated, and shown for non-imputed data.

Abbreviations: CES-D, Center for Epidemiologic Studies Depression Scale; HADS, Hospital Anxiety and Depression Scale; IQR, interquartile range; PSQI, Pittsburgh Sleep Quality Index; SD, standard deviation.

### Hierarchical clustering results

#### Optimal number of clusters

After visual inspection of the elbow method for items and participants separately (Figure 1 in the Supplementary Material) and assessing average silhouette scores for a *k*_i_ = 3–10 and *k*_p_ = 3–10 matrix (Figure 2 in the Supplementary Material), we identified the region of interest in the *k*_i_ × *k*_p_ space and explored cluster quality for potential item clusterings (*k*_i_ = 3–5) and participant clusterings (*k*_p_ = 3–5). This approach clearly favored a three-item clustering, but diverged on the optimal number of participant clusters inside a three-cluster item dataset (Table 2 in the Supplementary Material). Both on original HCA clusters and after *k*-means clustering (*k*-means clustering performed better than pam, data not shown), the silhouette approach indicated three participant clusters, while the between/total sum-of-squares ratio favored a five-cluster solution (Table 2 in the Supplementary Material). When we assessed both metrics based on ranking the middle ground 3 × 4 clustering solution scored the highest. Random resampling of participants and re-running HCA also indicated cluster stability, that is, items consistently co-occurred in the same cluster (Figure 3 in the Supplementary Material). Additionally, item clusters for male versus female participants showed no substantial differences compared to the total population (Table 3 and Figure 4 in the Supplementary Material).

#### Item clusters

**Figure 1.**
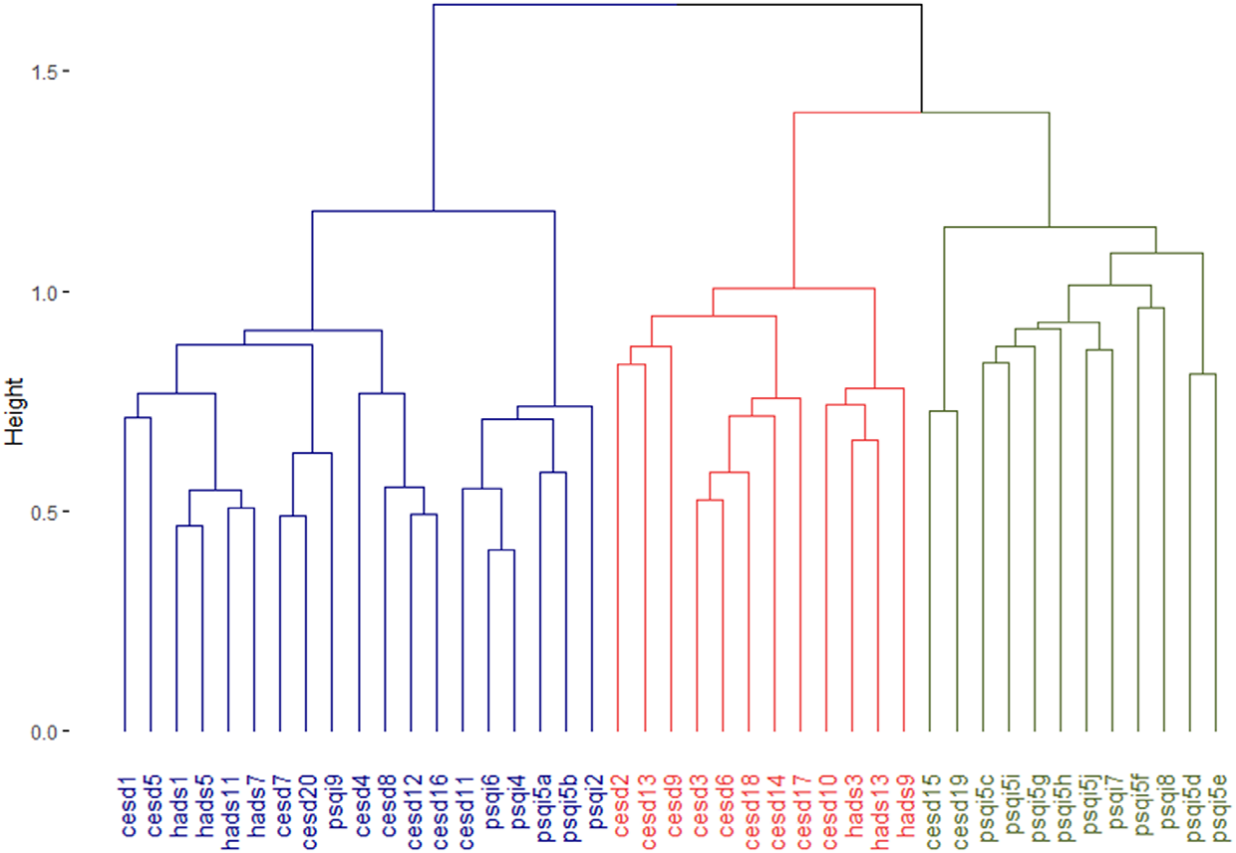
Dendrogram that represents the three-cluster solution of hierarchical clustering analysis on test items. Items that often co-occur cluster at lower levels (*y*-axis) in the dendrogram, while items that less often co-occur cluster only at higher level. The Jaccard index was used as the proximity measure and Ward’s method as the linkage criterion. Description of questionnaire items can be found in Table 3. CES-D, Center for Epidemiologic Studies Depression Scale; HADS, Hospital Anxiety and Depression Scale; PSQI, Pittsburgh Sleep Quality Index.

The item clusters are presented in Table 2 and Figure 1. The corresponding questions are added in Table 3. Cluster 1 (“mixed”) consisted of questions from all three questionnaires (nine CES-D, four HADS-A, and six PSQI items, mean endorsement: 6.8 out of 19 items) and represented items covering cognitive complaints, positive affect, restlessness, and sleep quantification. Cluster 2 (“depressed affect and nervousness”) consisted of mainly depression and anxiety questions (nine CES-D and three HADS-A items, mean endorsement: 1.6 out of 12 items), while cluster 3 (“troubled sleep and interpersonal problems”) consisted mainly of sleep quality questions and two items on social connection (2 CES-D and 10 PSQI items, mean endorsement: 1.0 out of 10 items). Cluster scores were moderately correlated (Spearman’s *ρ* = 0.31–0.48) except for the “mixed” and “depressed affect and nervousness” clusters (*ρ* = 0.66, Table 4 in the Supplementary Material). This is also shown in the correlation matrix of test items ordered by cluster membership (Figure 4 in the Supplementary Material). The median absolute deviation, which we normalized by number of items per cluster, was 0.23 for the “mixed” cluster, 0.12 for the “depressed affect and nervousness” cluster, and 0.15 for the “troubled sleep and interpersonal problems” cluster, indicating that overall symptom endorsement in the “mixed” cluster is most discriminatory across participants.

**Table 2.** The three-cluster solution of hierarchical clustering on test items in the discovery sample.

	Test items
“Mixed”	cesd1, cesd4, cesd5, cesd7, cesd8, cesd11, cesd12, cesd16, cesd20, hads1, hads5, hads7, hads11, psqi2, psqi4, psqi5a, psqi5b, psqi6, psqi9
“Depressed affect and nervousness”	cesd2, cesd3, cesd6, cesd9, cesd10, cesd13, cesd14, cesd17, cesd18, hads3, hads9, hads13
“Troubled sleep and interpersonal problems”	cesd15, cesd19, psqi5c, psqi5d, psqi5e, psqi5f, psqi5g, psqi5h, psqi5i, psqi5j, psqi7, psqi8

*Note*: Description of questionnaire items can be found in Table 3.

Abbreviations: CES-D, Center for Epidemiologic Studies Depression Scale; HADS, Hospital Anxiety and Depression Scale; PSQI, Pittsburgh Sleep Quality Index.

**Table 3.** The three-cluster solution of hierarchical clustering on test items.

	Questionnaire item
**“Mixed”**	
Cesd1	I was bothered by things that usually do not bother me.
Cesd4	I felt that I was just as good as other people.
Cesd5	I had trouble keeping my mind on what I was doing.
Cesd7	I felt that everything I did was an effort.
Cesd8	I felt hopeful about the future.
Cesd11	My sleep was restless.
Cesd12	I was happy.
Cesd16	I enjoyed life.
Cesd20	I could not get “going.”
Hads1	I feel tense or “wound up.”
Hads5	Worrying thoughts go through my mind.
Hads7	I can sit at ease and feel relaxed.
Hads11	I feel restless as if I have to be on the move
Psqi2	How long (in minutes) has it usually take you to fall asleep each night?
Psqi4	How many hours of actual sleep did you get at night?
Psqi5a	How often have you had trouble sleeping because you cannot get to sleep within 30 min?
Psqi5b	How often have you had trouble sleeping because you wake up in the middle of the night or early morning?
Psqi6	How would you rate your sleep quality overall?
Psqi9	How much of a problem has it been for you to keep up enough enthusiasm to get things done?
**“Depressed affect and nervousness”**	
Cesd2	I did not feel like eating; my appetite was poor.
Cesd3	I felt that I could not shake off the blues even with help from my family or friends.
Cesd6	I felt depressed.
Cesd9	I thought my life had been a failure.
Cesd10	I felt fearful.
Cesd13	I talked less than usual.
Cesd14	I felt lonely.
Cesd17	I had crying spells.
Cesd18	I felt sad.
Hads3	I get a sort of frightened feeling like something awful is about to happen.
Hads9	I get a sort of frightened feeling like “butterflies in the stomach.”
Hads13	I get sudden feelings of panic.
**“Troubled sleep and interpersonal problems”**	
Cesd15	People were unfriendly.
Cesd19	I felt that people dislike me.
Psqi5c	How often have you had trouble sleeping because you have to get up to use the bathroom?
Psqi5d	How often have you had trouble sleeping because you cannot breathe comfortably?
Psqi5e	How often have you had trouble sleeping because you cough or snore loudly?
Psqi5f	How often have you had trouble sleeping because you feel too cold?
Psqi5g	How often have you had trouble sleeping because you feel too hot?
Psqi5h	How often have you had trouble sleeping because you had bad dreams?
Psqi5i	How often have you had trouble sleeping because you have pain?
Psqi5j	How often have you had trouble sleeping because of other reasons?
Psqi7	How often have you taken medicine (prescribed or “over the counter”) to help you sleep?
Psqi8	How often have you had trouble staying awake while driving, eating meals, or engaging in social activity?

Abbreviations: CES-D, Center for Epidemiologic Studies Depression Scale; HADS, Hospital Anxiety and Depression Scale; PSQI, Pittsburgh Sleep Quality Index.

#### Participant groups

**Figure 2.**
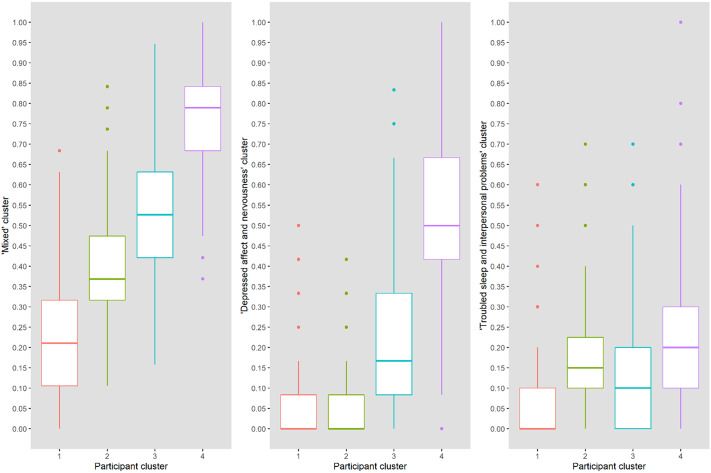
Participant groups and their scores on each item cluster. The boxplots show the aggregate cluster scores (*y*-axis) of the four identified participant groups (*x*-axis) on the three clusters of test items. Item cluster 1, mixed; item cluster 2, depressed affect and nervousness; item cluster 3, troubled sleep and interpersonal problems.

The four participant groups that are presented in the dendrogram (Figure 5 in the Supplementary Material) represent groups of participants that were most similar in our data and may therefore share underlying psychopathology or circumstances. Participants in the first group, who were more often higher educated (23.5%) and living with partner (74.5%), scored lowest on all item clusters (Figure 2 and Table 5 in the Supplementary Material). The second group scored higher on the “mixed” cluster than the first, and also indicated symptoms of “troubled sleep and interpersonal problems.” In general, items of the “mixed” cluster were most common and were indicated by all participants groups. The third and fourth group also indicated items of the “depressed affect and nervousness” and the “troubled sleep and interpersonal problems” clusters. Participants in the fourth group, who were more often female (78.3%), living alone (45.9%), and current smokers (26.1%), scored highest on all item clusters (Table 5 in the Supplementary Material). Within groups, cluster score correlations were low to moderate (*ρ* < 0.35, Table 4 in the Supplementary Material).

#### Replication of hierarchical clustering analyses

Regarding item clusters, replication in an independent sample (*N* = 3,005) confirmed that a three-cluster solution was preferred. Item clusters, presented in Table 6 and Figure 6 in the Supplementary Material, were highly similar. Four items of the “troubled sleep and interpersonal problems” moved to other clusters. Specifically, the two items that we referred to as “interpersonal problems” now belonged to the “depressed affect and nervousness cluster,” but the clustering results of the discovery sample already showed that those items only clustered at a relatively high level to other items.

Regarding participant groups, we again yielded a four-group solution (Table 1 and Figure 7 in the Supplementary Material). We observed one group that scored lowest on all clusters, and one group that scored highest on all clusters. Similar to the discovery sample, items from the “mixed” clusters were most common and indicated by all participant groups.

### Gaussian finite mixture modeling of participants with a formal diagnosis

**Figure 3.**
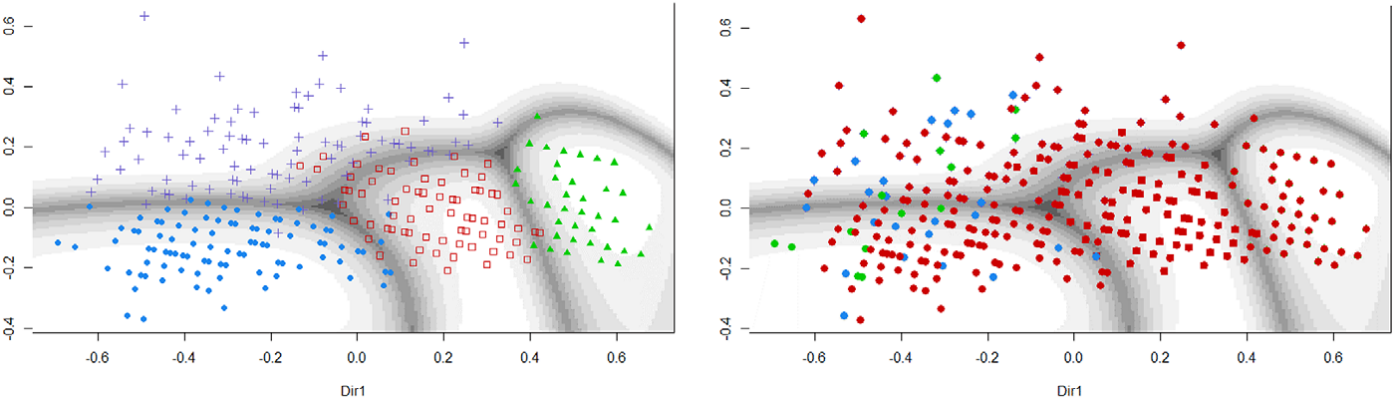
Clustering of participants with a known diagnosis of depression and/or anxiety. The left panel shows the contourplot that represents the cluster solution within a subsample of participants with a known diagnosis of depression (i.e., major depressive disorder of dysthymia, *N* = 40), anxiety disorder (*N* = 352), or both (*N* = 21), based on MClust Gaussian finite mixture modeling. The right panel indicates original diagnosis of participants within these data-driven clusters. Red, anxiety; blue, depression; green, comorbid.

For a subgroup of participants with a known diagnosis of depression, anxiety, or both, Gaussian finite mixture modeling suggested a four-group solution (based on BIC) with a covariance structure resulting in clusters with variable volume, equal shape, and variable orientation (VEV). The obtained contour plot and corresponding cluster loadings are presented in Figure 3 and Table 7 in the Supplementary Material. When looking at the distribution of diagnoses across these data-driven clusters, depression, and comorbid diagnoses were mostly restricted to groups 1 and 4, while patients with an anxiety diagnosis were spread out across all groups (Figure 3). Undersampling to achieve a dataset balanced between depression and anxiety diagnoses yielded a small sample (*N* = 101) and a cluster solution that was again informative on general severity but not on type of symptoms (data not shown).

## Discussion

Based on data from a population-based study of middle-aged and older adults, we identified three item clusters, representing “mixed” symptoms, “depressed affect and nervousness” and “troubled sleep and interpersonal problems.” Data-driven clusters of items assessing symptoms of depression, anxiety, and poor sleep differed substantially from the ordering of symptoms as presented in these questionnaires largely based on diagnostic criteria.

The “mixed” item cluster represented items that indicated a low positive affect, cognitive complaints, restlessness, and more quantifiable aspects of sleep. Clustering of symptoms on sleep and energy was previously reported in a clinical sample, yet our “mixed” cluster incorporates other symptoms, most distinctly a low positive affect [30]. Low positive affect was suggested to be specific for depressive symptoms, also based on a clinical sample, suggesting that this feature could be used to differentiate from anxiety symptoms [31]. Our findings indicate that this does not hold on a subclinical level in a population-based setting, as we have found low positive affect to be clustered with items related to both anxiety and sleep quality. Also, the “mixed” cluster is not fully in line with previous studies using factor analyses, as shown by the HiTOP framework where these symptoms are mostly presented in the “distress” subfactor including symptoms of general depression and anxiety [8, 9]. As symptoms in the “mixed” cluster were most common in our population-based sample, this item cluster potentially reflects complaints that are common subclinical symptoms and are not specific to one disorder. Also, these symptoms might be most informative to differentiate between participants as most variance was found within this cluster.

The “depressed affect and nervousness” cluster consisted of items that represented low mood, including all items of the depressed affect subscale of the CES-D, and some anxiety items, which seem to indicate nervousness or a level of general anxiety. These findings are in line with results of Paykel (1971) and the tripartite model of Clark and Watson (1991) based on clinical populations, suggesting that depression and anxiety share common features in general psychological distress and low mood disorders [31, 32]. Also, these results agree with the HiTOP framework, as both general depression and general anxiety are presented in the “distress” subfactor within the “internalizing” spectrum [8, 9]. The clustering of these items on both a subclinical and clinical level might very well contribute to the common comorbidity of depressive and anxiety disorders.

Finally, the “troubled sleep and interpersonal problems” cluster contained PSQI and CES-D items that represented sleep disturbances and interpersonal problems. These subclusters do not seem to be directly relatable, and the dendrogram also showed that these subclusters merged at a relatively high level, suggesting more sub-cluster closeness than shared similarity. Also, the clustering between symptoms of sleep disturbances and interpersonal problems was not confirmed in the replication sample.

Using a similar hierarchical clustering approach on participants, we have identified four groups that showed differential patterns of psychiatric symptom endorsement aggregated in the aforementioned item clusters. In line with previous work, these groups could party, but not fully, be explained by overall symptom severity [14]. Interestingly, our results in a nonclinical population might give important insights in severity of symptoms and consequently, for diagnosis. Symptoms of low positive affect, cognitive complaints, restlessness, and poor general sleep quality were most common in our study population and did not necessarily co-occur with symptoms of depressed affect and general anxiety, as was suggested by previous, mostly clinical, studies [8, 9]. Only when these “mixed” symptoms were severe to some extent, it was likely that participants also experienced symptoms of “depressed affect and nervousness.” Ultimately, this knowledge could help in the development of screening and targeted prevention strategies.

Altogether, our data-driven approach has shown three clusters of test items that showed substantial mixing of existing subscales, arguing against an interpretation where the scales under investigation are indexing underlying phenomena directly and independently. Our findings suggested subclinical symptom clusters to be different from clusters in clinical samples, indicating specific subclinical symptoms that might ultimately be useful for earlier detection of more severe symptoms. By identifying subgroups with similar patterns of (sub)clinical symptoms, prevention and treatment strategies could be developed aimed at reducing specific symptom groupings, instead of being targeted at the diagnostic level [33]. Using large datasets, clustering of symptoms as presented in our study could be validated and extended, specifically, these clusters need to be validated against external variables such as use of mental health services. Also, the identified symptom clusters could be related to other health indicators and ultimately offer possibility to gain more insight in the biology underlying psychiatric symptoms.

The strengths of this study lie in the large population-based discovery and replication samples combined with unsupervised clustering methods, a benefit over more opaque deep learning models in medical settings that require explainable algorithms. However, several limitations should be taken into account when interpreting the results. First, the cross-sectional nature of the data implied that we could not investigate trajectories or episodes of psychiatric symptoms. Second, we used one specific questionnaire for each phenotype, while other diagnostic tools could have given other insights or results, validation across measurements is warranted. Third, a diagnostic measure of sleep–wake disorders was not available and therefore, we could base the undersampling on participants with a known diagnosis only on depressive and anxiety disorders. Fourth, both hierarchical and *k*-means clustering do not have strict criteria to define the optimal number of clusters. However, using cluster quality metrics that are agnostic to the number of features in the dataset (average silhouette width and the proportion of between over total-sum-of-squares) and resampling methods, we could decide on the optimal solution for this sample and assess its explanatory power over a random clustering method. Fifth, participants were sampled from one specific neighborhood in Rotterdam and were only above a certain age, resulting in a sample that is not fully representative of the full Dutch population. The low percentage of participants fulfilling diagnostic criteria for depression might be explained by the older age and by the assessment of prevalent depression only (depression at time of assessment, rather than over a certain time period or lifetime), and agrees to previous studies with a similar sampling strategy [34]. Finally, in this study, we assessed each test item or symptom as equally important. However, there might be specific symptoms that “deserve” more weight, such as the core symptoms of depression. In future studies, both theory-driven and data-driven ways could be explored to overcome this, for example by modeling latent factors that estimate weights for individual symptoms within symptom clusters.

## Conclusion

Our findings emphasize that clustering of subclinical psychiatric symptoms on a population level differs substantially from clusters as reported in diagnostic criteria. This underlines the heterogeneous nature of symptom profiles across the population. Also, this might explain the high rates of comorbidity of psychiatric diagnoses once symptoms reach a clinical level. Our findings further emphasize the need for more overarching approaches, looking beyond current categorizations of symptoms based on diagnostic criteria, based on individual (sub)clinical symptoms profiles.

## Data Availability

Data can be obtained upon request. Requests should be directed toward the management team of the Rotterdam Study (secretariat.epi@erasmusmc.nl), which has a protocol for approving data requests. Because of restrictions based on privacy regulations and informed consent of the participants, data cannot be made freely available in a public repository.

## Supplementary material

For supplementary material accompanying this paper visit http://doi.org/10.1192/j.eurpsy.2023.13. click here to view supplementary material
