# Supplementary material for: Uncovering psychiatric phenotypes using unsupervised machine learning: A data-driven symptoms approach
Source: Eur Psychiatry. 2023 Feb 21;66(1):e27. doi: 10.1192/j.eurpsy.2023.13 (PMC10044296; doi:10.1192/j.eurpsy.2023.13)
Supplement: Supplementary file 1 [file S0924933823000135sup001.docx]

**Supplemental material**

**Appendix, Table 1: Characteristics of the replication sample, total population and per participant group**

|  | **Total**  **(N=3005)** | **Participant group 1**  **(N=1731)** | **Participant group 2**  **(N=511)** | **Participant group 3**  **(N=398)** | **Participant group 4**  **(N=365)** |
| --- | --- | --- | --- | --- | --- |
| Age (years) | 57.3 (11.4) | 57.2 (11.7) | 58.0 (11.5) | 57.6 (11.5) | 56.2 (10.2) |
| Sex  Female | 1723 (57.3%) | 874 (50.5%) | 329 (64.4%) | 273 (68.6%) | 247 (67.7%) |
| Educational level  Primary  Lower  Further / intermediate  Higher | 208 (6.9%)  737 (24.5%)  1120 (37.3%)  914 (30.4%) | 107 (6.2%)  419 (24.2%)  619 (35.8%)  576 (33.3%) | 39 (7.6%)  123 (24.1%)  201 (39.3%)  143 (28.0%) | 29 (7.3%)  99 (24.9%)  154 (38.7%)  110 (27.6%) | 33 (9.0%)  96 (26.3%)  146 (40.0%)  85 (23.3%) |
| Marital status  Living with partner  Living alone | 2059 (68.5%)  942 (31.3%) | 1239 (71.6%)  489 (28.2%) | 359 (70.3%)  152 (29.7%) | 249 (62.6%)  148 (37.2%) | 212 (58.1%)  153 (41.9%) |
| Paid employment  Yes | 1856 (61.8%) | 1132 (65.4%) | 324 (63.4%) | 225 (56.5%) | 175 (47.9%) |
| Smoking  Current smoker  Current non-smoker | 576 (19.2%)  2426 (80.7%) | 322 (18.6%)  1407 (81.3%) | 82 (16.0%)  429 (84.0%) | 74 (18.6%)  324 (81.4%) | 98 (26.8%)  266 (72.9%) |

Data are presented as mean(SD) or N(%) unless otherwise indicated, and shown for non-imputed data. SD = Standard Deviation.

**Appendix, Table 2: Cluster quality metrics for possible combinations of item and participant clusters**

|  | **Average silhouette width** | | |  | **Between/total sum-of-squares** | | |
| --- | --- | --- | --- | --- | --- | --- | --- |
| **Results based on HCA** | | | | | | | |
|  | **3** | **4** | **5** |  | **3** | **4** | **5** |
| **3** | 0.46 | 0.24 | 0.21 | **3** | 0.58 | 0.63 | 0.64 |
| **4** | 0.47 | 0.25 | 0.23 | **4** | 0.54 | 0.58 | 0.60 |
| **5** | 0.47 | 0.28 | 0.26 | **5** | 0.54 | 0.58 | 0.60 |
| **Results after running kmeans** | | | | | | | |
|  | **3** | **4** | **5** |  | **3** | **4** | **5** |
| **3** | 0.64 | 0.55 | 0.46 | **3** | 0.76 | 0.81 | 0.84 |
| **4** | 0.55 | 0.47 | 0.46 | **4** | 0.66 | 0.71 | 0.75 |
| **5** | 0.55 | 0.47 | 0.46 | **5** | 0.66 | 0.71 | 0.75 |

Columns refer to number of item clusters, rows to number of participant clusters.

| **Clusters total population** | **Women** | **Men** |
| --- | --- | --- |
| **‘Mixed’** |  |  |
| Cesd1 | 1 | 1 |
| Cesd4 | 1 | 1 |
| Cesd5 | 1 | 1 |
| Cesd7 | 1 | 1 |
| Cesd8 | 1 | 1 |
| Cesd11 | 1 | 1 |
| Cesd12 | 1 | 1 |
| Cesd16 | 1 | 1 |
| Cesd20 | 1 | 1 |
| Hads1 | 1 | 1 |
| Hads5 | 1 | 1 |
| Hads7 | 1 | 1 |
| Hads11 | 1 | 1 |
| Psqi2 | 1 | 1 |
| Psqi4 | 1 | 1 |
| Psqi5a | 1 | 1 |
| Psqi5b | 1 | 1 |
| Psqi6 | 1 | 1 |
| Psqi9 | 1 | 1 |
| **‘Depressed affect and nervousness’** |  |  |
| Cesd2 | 2 | 3 |
| Cesd3 | 2 | 2 |
| Cesd6 | 2 | 2 |
| Cesd9 | 2 | 2 |
| Cesd10 | 2 | 2 |
| Cesd13 | 2 | 2 |
| Cesd14 | 2 | 2 |
| Cesd17 | 2 | 2 |
| Cesd18 | 2 | 2 |
| Hads3 | 2 | 2 |
| Hads9 | 2 | 2 |
| Hads13 | 2 | 2 |
| **‘Troubled sleep and interpersonal problems’** |  |  |
| Cesd15 | 3 | 3 |
| Cesd19 | 3 | 3 |
| Psqi5c | 3 | 1 |
| Psqi5d | 3 | 3 |
| Psqi5e | 3 | 3 |
| Psqi5f | 3 | 3 |
| Psqi5g | 3 | 3 |
| Psqi5h | 3 | 3 |
| Psqi5i | 3 | 3 |
| Psqi5j | 3 | 1 |
| Psqi7 | 3 | 3 |
| Psqi8 | 3 | 3 |

**Appendix, Table 3: The 3-cluster solution of hierarchical clustering on test items for men and women separately, compared to the total population**

CES-D = Center for Epidemiologic Studies Depression Scale, HADS = Hospital Anxiety and Depression scale, PSQI = Pittsburgh Sleep Quality Index.

| **Correlations between item clusters, total sample** | | | |
| --- | --- | --- | --- |
|  | ‘Mixed’ | ‘Depressed affect and nervousness’ | ‘Troubled sleep and interpersonal problems’ |
| ‘Mixed’ | 1.00 |  |  |
| ‘Depressed affect and nervousness’ | 0.66 | 1.00 |  |
| ‘Troubled sleep and interpersonal problems’ | 0.48 | 0.31 | 1.00 |
| **Correlations between item clusters, in each participant group separately** | | | |
| Group 1 | ‘Mixed’ | ‘Depressed affect and nervousness’ | ‘Troubled sleep and interpersonal problems’ |
| ‘Mixed’ | 1.00 |  |  |
| ‘Depressed affect and nervousness’ | 0.14 | 1.00 |  |
| ‘Troubled sleep and interpersonal problems’ | 0.32 | -0.01 | 1.00 |
| Group 2 |  |  |  |
| ‘Mixed’ | 1.00 |  |  |
| ‘Depressed affect and nervousness’ | 0.35 | 1.00 |  |
| ‘Troubled sleep and interpersonal problems’ | 0.29 | 0.10 | 1.00 |
| Group 3 |  |  |  |
| ‘Mixed’ | 1.00 |  |  |
| ‘Depressed affect and nervousness’ | 0.33 | 1.00 |  |
| ‘Troubled sleep and interpersonal problems’ | 0.18 | 0.14 | 1.00 |
| Group 4 |  |  |  |
| ‘Mixed’ | 1.00 |  |  |
| ‘Depressed affect and nervousness’ | 0.25 | 1.00 |  |
| ‘Troubled sleep and interpersonal problems’ | 0.23 | 0.10 | 1.00 |

**Appendix, Table 4: Spearman correlations between clusters, in total sample and within participant groups**

**Appendix, Table 5: Characteristics of the study sample per participant group in discovery sample.**

|  | **Participant group 1**  **(N=3859, 58%)** | **Participant group 2**  **(N=752, 11%)** | **Participant group 3**  **(N=1309, 20%)** | **Participant group 4**  **(N=682, 10%)** |
| --- | --- | --- | --- | --- |
| Age (years) | 69.1 (9.1) | 71.2 (9.2) | 69.5 (9.9) | 69.5 (10.0) |
| Sex  Female | 1868 (48.4%) | 547 (72.7%) | 867 (66.2%) | 534 (78.3%) |
| Educational level  Primary  Lower  Further / intermediate  Higher | 298 (7.7%)  1439 (37.3%) 1177 (30.5%)  905 (23.5%) | 78 (10.4%)  328 (43.6%)  200 (26.6%)  136 (18.1%) | 126 (9.6%)  576 (44.0%)  351 (26.8%)  246 (18.8%) | 77 (11.3%)  309 (45.3%)  187 (27.4%)  101 (14.8%) |
| Marital status  Living with partner  Living alone | 2874 (74.5%)  977 (25.3%) | 491 (65.3%)  261 (34.7%) | 819 (62.6%)  490 (37.4%) | 368 (54.0%)  313 (45.9%) |
| Paid employment  Yes | 874 (22.6%) | 122 (16.2%) | 256 (19.6%) | 111 (16.3%) |
| Smoking  Current smoker  Current non-smoker | 492 (12.7%)  3350 (86.8%) | 57 (7.6%)  693 (92.2%) | 178 (13.6%)  1130 (86.3%) | 178 (26.1%)  560 (82.1%) |

Data are presented as mean(SD) or N(%) unless otherwise indicated, and shown for non-imputed data. SD = Standard Deviation.

|  | **Test items** |
| --- | --- |
| ‘Mixed’ | cesd1, cesd4, cesd5, cesd7, cesd8, cesd11, cesd12, cesd16, cesd20, hads1, hads5, hads7, hads11, psqi2, psqi4, psqi5a, psqi5b, psqi5c, psqi5j, psqi6, psqi9 |
| ‘Depressed affect and nervousness’ | cesd2, cesd3, cesd6, cesd9, cesd10, cesd13, cesd14, cesd15, cesd17, cesd18, cesd19, hads3, hads9, hads13 |
| ‘Troubled sleep’ | psqi5d, psqi5e, psqi5f, psqi5g, psqi5h, psqi5i, psqi7, psqi8 |

**Appendix, Table 6: The 3-cluster solution of hierarchical clustering on test items in the replication sample**

CES-D = Center for Epidemiologic Studies Depression Scale, HADS = Hospital Anxiety and Depression scale, PSQI = Pittsburgh Sleep Quality Index. Description of questionnaire items can be found in Appendix Table 4.

**Appendix, Table 7: Cluster loadings of Gaussian Finite Mixture Modelling using mclust**

|  | **Dir1** | **Dir2** | **Dir3** |
| --- | --- | --- | --- |
| Item cluster 1 | -0.74384 | 0.23401 | -0.72690 |
| Item cluster 2 | -0.64284 | -0.37268 | 0.50308 |
| Item cluster 3 | 0.18291 | 0.89797 | 0.46747 |

*
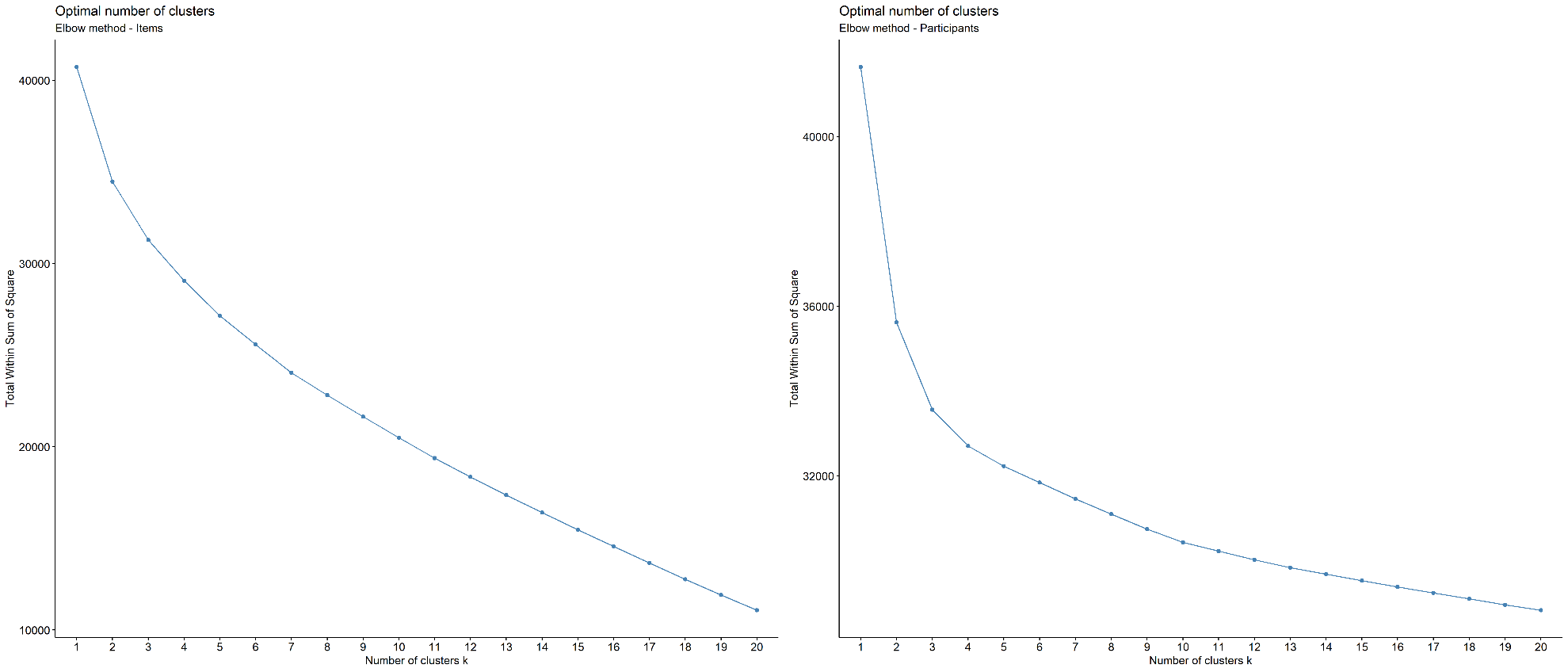
*

**Appendix, Figure 1.** Graphical representation of the elbow method for items and participants separately.


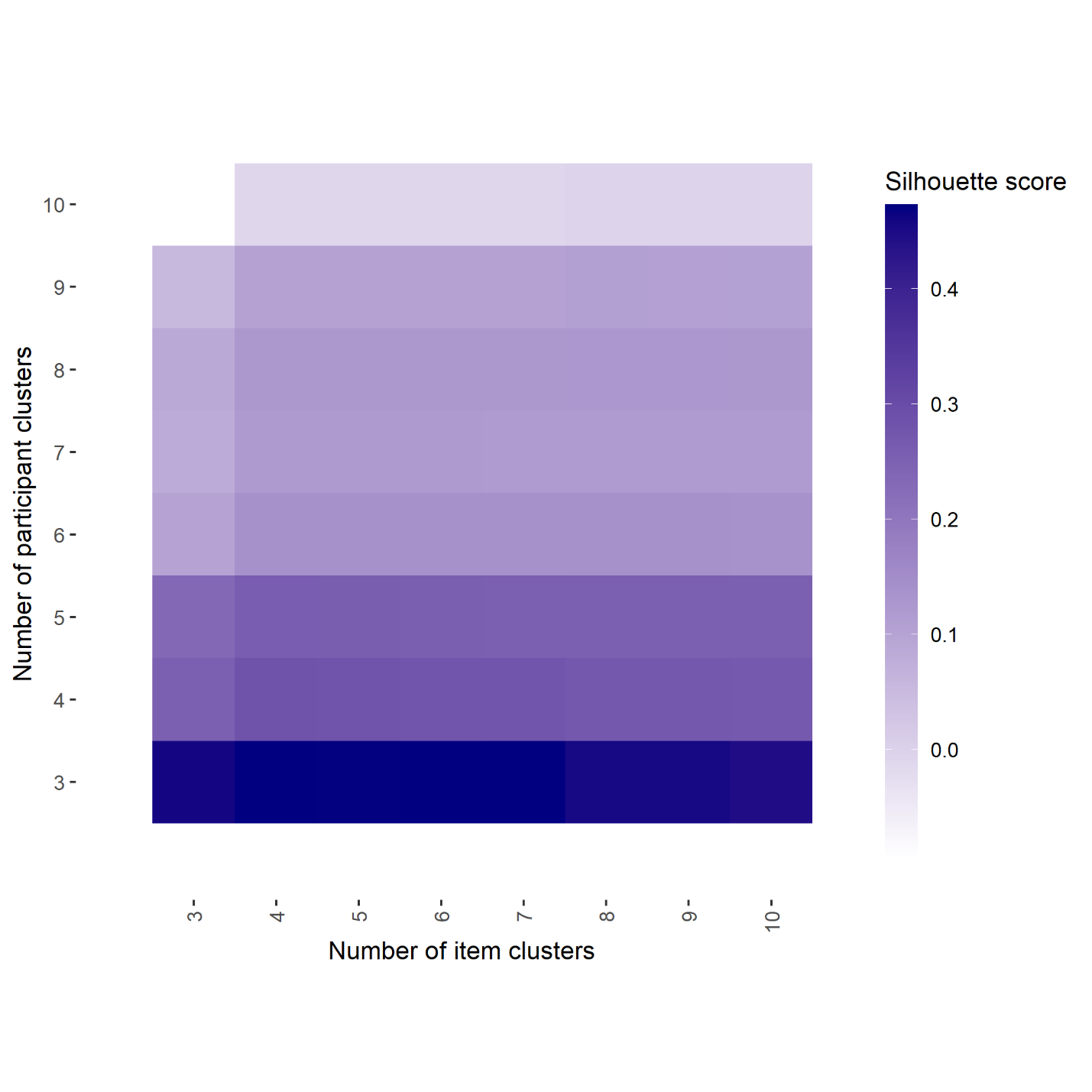


**Appendix, Figure 2.** Heatmap that shows silhouette scores for possible combinations of *k*_i_ item cluster and *k*_p_ participant clusters.

*
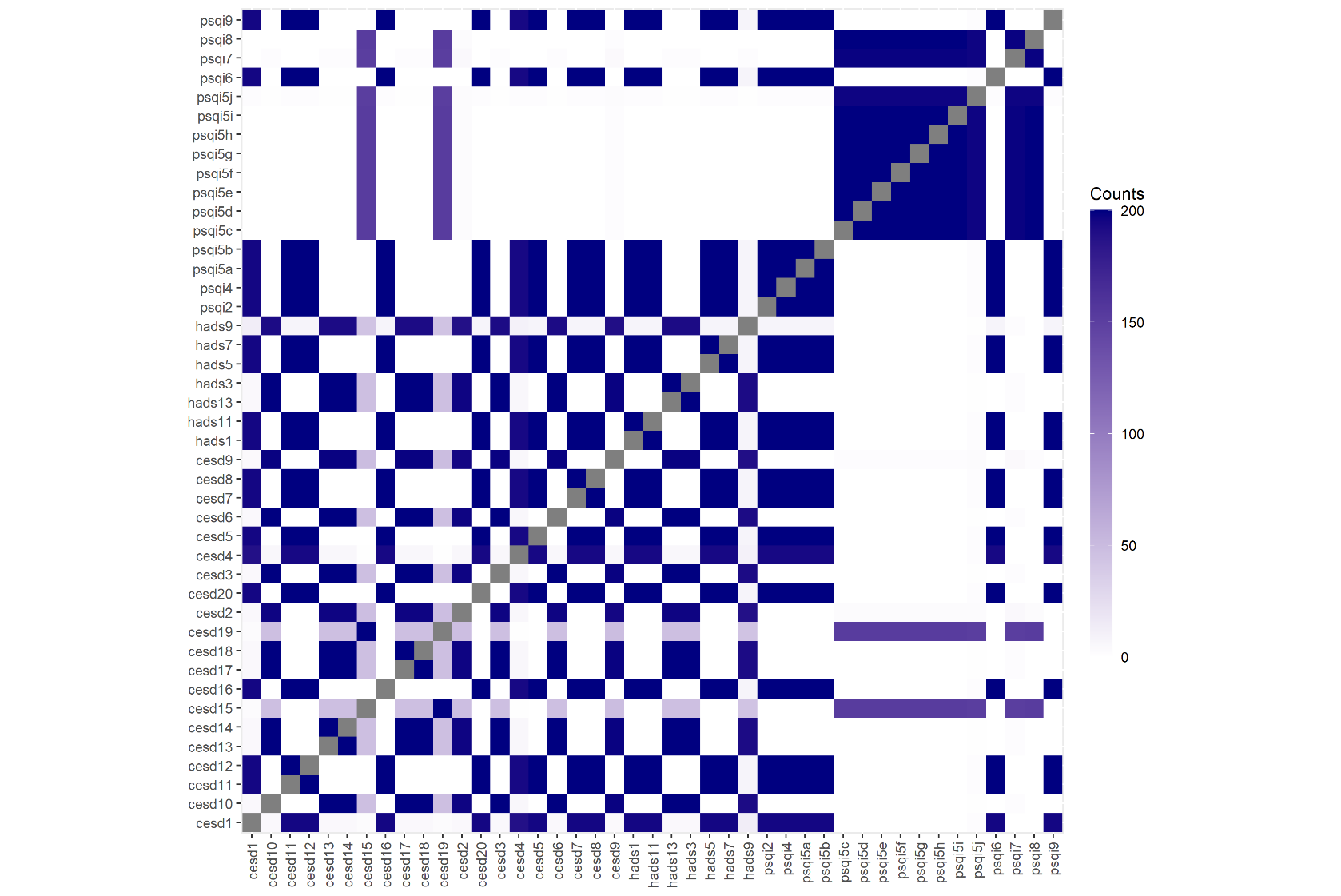
* **Appendix, Figure 3.** Heatmap that indicates how often two items occurred in the same cluster when randomly resampling and clustering the data using hierarchical clustering analysis 200 times.
Description of questionnaire items can be found in Appendix Table 4.


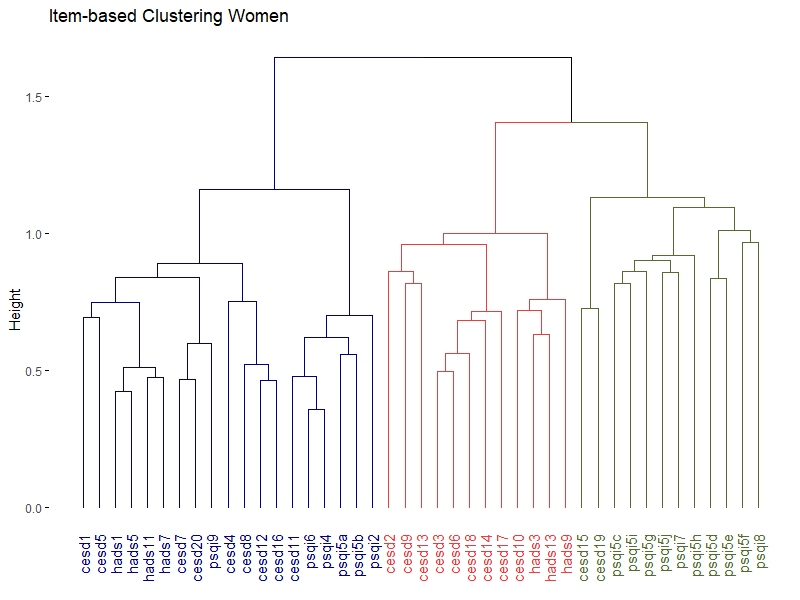

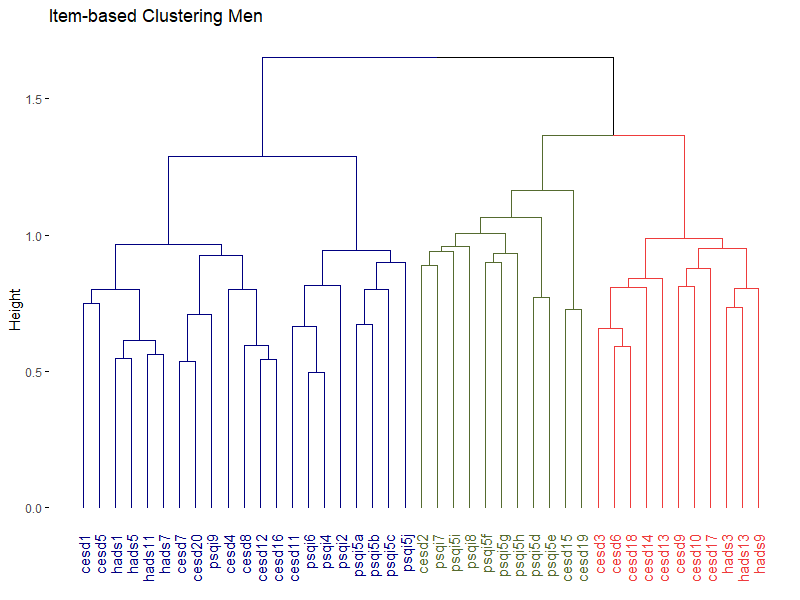

**Appendix, Figure 4.** Dendrograms that represents the 3-cluster solution of hierarchical clustering analysis on test for men and women separately. Items that often co-occur cluster at lower levels in the dendrogram, while items that less often co-occur cluster only at higher level. Jaccard index was used as the proximity measure and Ward’s method as the linkage criterion. CES-D = Center for Epidemiologic Studies Depression Scale, HADS = Hospital Anxiety and Depression scale, PSQI = Pittsburgh Sleep Quality Index. Description of questionnaire items can be found in Appendix Table 4.


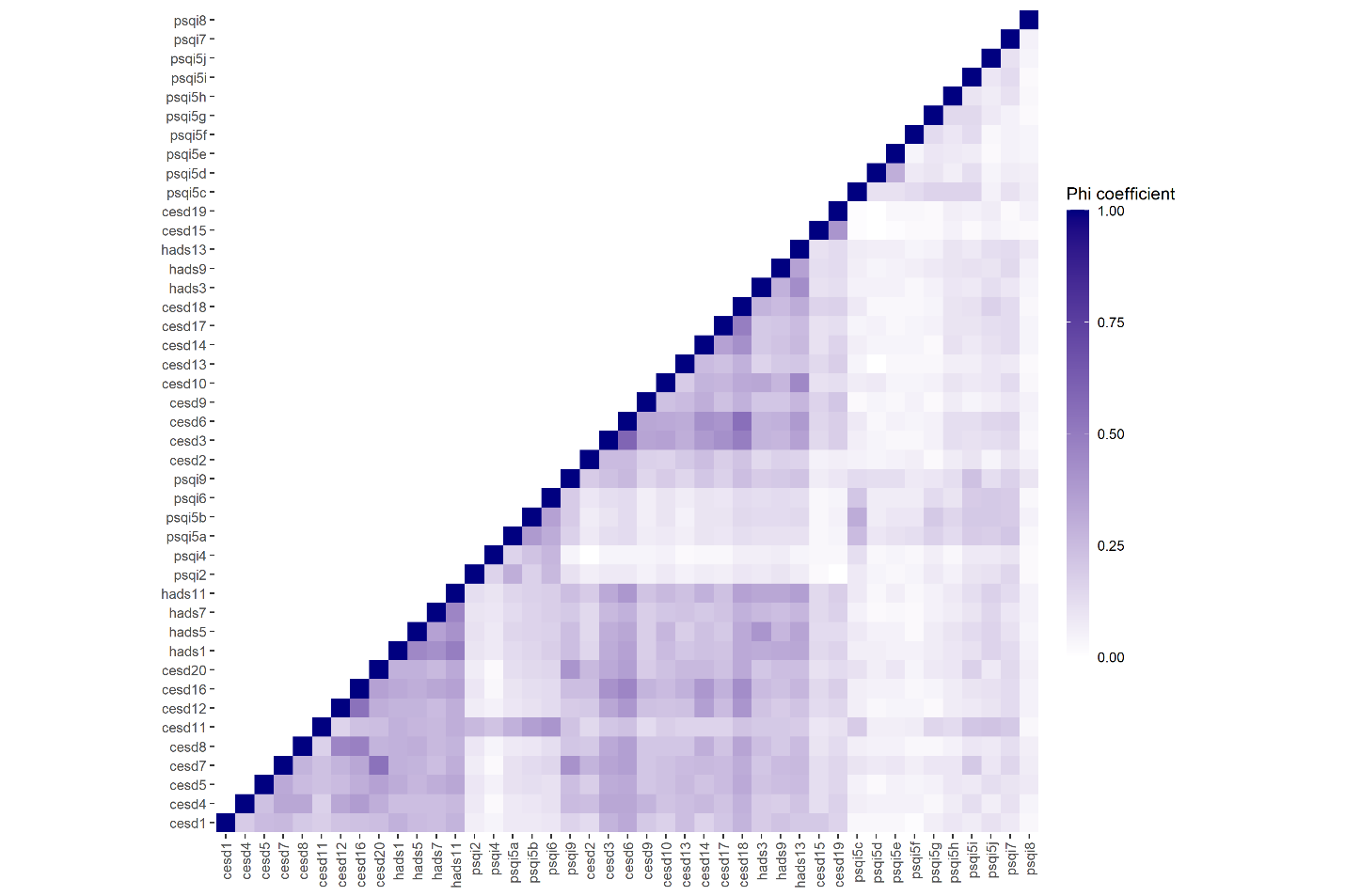


**Appendix, Figure 4.** Heatmap that indicates correlations between test items ordered by cluster membership.
Description of questionnaire items can be found in Appendix Table 4. Phi coefficient was used as item variables were dichotomous.

**Appendix, Figure 5.** Dendrogram that represents the 4-cluster solution of hierarchical clustering analysis on participants. Participants that were more similar based on their data cluster at lower levels in the dendrogram, while participants who were less similar based on their data cluster only at higher level. Simple Matching Coefficient was used as the proximity measure and Ward’s method as the linkage criterion.


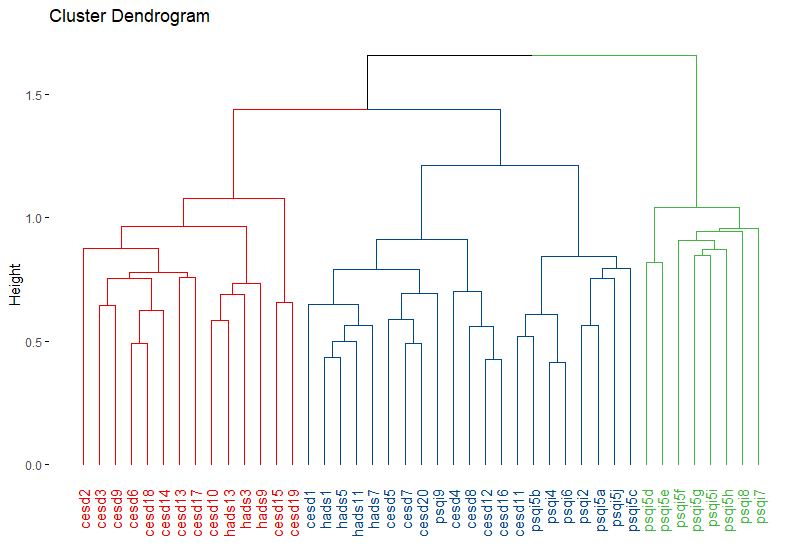
**Appendix, Figure 6.** Dendrogram that represents the 3-cluster solution of hierarchical clustering analysis on test items in the replication sample. Items that often co-occur cluster at lower levels (y-axis) in the dendrogram, while items that less often co-occur cluster only at higher level. The Jaccard index was used as the proximity measure and Ward’s method as the linkage criterion.
CES-D = Center for Epidemiologic Studies Depression Scale, HADS = Hospital Anxiety and Depression scale, PSQI = Pittsburgh Sleep Quality Index. Description of questionnaire items can be found in Appendix Table 4.


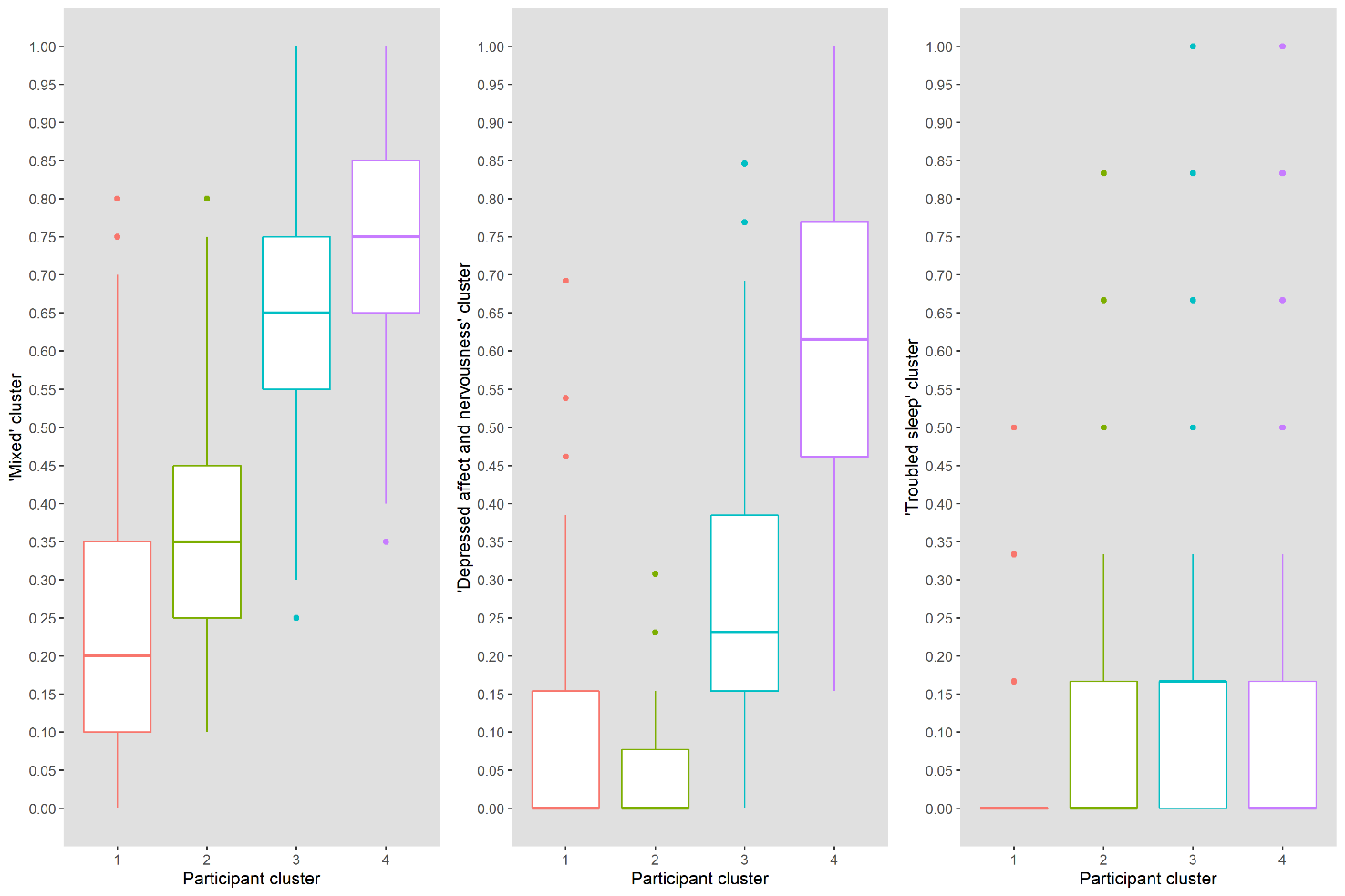


**Appendix, Figure 7.** Participant groups and their scores on each item cluster in the replication sample. The boxplots show the aggregate cluster scores (y-axis) of the four identified participant groups (x-axis) on the three clusters of test items.
Item cluster 1 = ‘Mixed’, item cluster 2 = ‘Depressed affect and nervousness’, item cluster 3 = ‘Troubled sleep and interpersonal problems’.
